# Comparative assessment of passive surveillance in disease-free and endemic situation: Example of *Brucella melitensis *surveillance in Switzerland and in Bosnia and Herzegovina

**DOI:** 10.1186/1746-6148-4-52

**Published:** 2008-12-22

**Authors:** Daniela C Hadorn, Sabina Seric Haracic, Katharina DC Stärk

**Affiliations:** 1 Federal Veterinary Office, Schwarzenburgstrasse 155, 3097 Bern-Liebefeld, Switzerland; 2 Veterinary Faculty, Zmaja od Bosne, 71 000 Sarajevo, Bosnia and Herzegovina; 3 Royal Veterinary College, Hawkshead Lane, North Mymms, Hertfordshire, AL97TA, UK

## Abstract

**Background:**

Globalization and subsequent growth in international trade in animals and animal products has increased the importance of international disease reporting. Efficient and reliable surveillance systems are needed in order to document the disease status of a population at a given time. In this context, passive surveillance plays an important role in early warning systems. However, it is not yet routinely integrated in the assessment of disease surveillance systems because different factors like the disease awareness (DA) of people reporting suspect cases influence the detection performance of passive surveillance. In this paper, we used scenario tree methodology in order to evaluate and compare the quality and benefit of abortion testing (ABT) for *Brucella melitensis *(*Bm*) between the disease free situation in Switzerland (CH) and a hypothetical disease free situation in Bosnia and Herzegovina (BH), taking into account DA levels assumed for the current endemic situation in BH.

**Results:**

The structure and input parameters of the scenario tree were identical for CH and BH with the exception of population data in small ruminants and the DA in farmers and veterinarians. The sensitivity analysis of the stochastic scenario tree model showed that the small ruminant population structure and the DA of farmers were important influential parameters with regard to the unit sensitivity of ABT in both CH and BH. The DA of both farmers and veterinarians was assumed to be higher in BH than in CH due to the current endemic situation in BH. Although the same DA cannot necessarily be assumed for the modelled hypothetical disease free situation as for the actual endemic situation, it shows the importance of the higher vigilance of people reporting suspect cases on the probability that an average unit processed in the ABT-component would test positive.

**Conclusion:**

The actual sensitivity of passive surveillance approaches heavily depends on the context in which they are applied. Scenario tree modelling allows for the evaluation of such passive surveillance system components under assumed disease free situation. Despite data gaps, this is a real opportunity to compare different situations and to explore consequences of changes that could be made.

## Background

Globalization and subsequent growth in international trade in animals and animal products has increased the importance of international disease reporting. Efficient and reliable surveillance systems are the basis for reliable disease reporting. The Agreement on the Application of Sanitary and Phytosanitary Measures (SPS Agreement) of the World Trade Organization [1] is a central document for international free trade and refers to the World Organization for Animal Health (OIE) as the organization responsible for setting international animal health standards. The quality of surveillance systems is crucial in order to assess and document the disease situation in a country or region. According to the OIE, a surveillance system for an infectious animal disease is defined as a method of surveillance that may include one or more component activities that generates information on the health, disease or zoonosis status of animal populations [2]. In general, such component activities or surveillance system components (SSCs) may be based on two different surveillance approaches, i.e. active and passive surveillance. Active surveillance is described by Lilienfeld & Stolley [3] as the regular periodic collection of samples or case reports by veterinary health authorities. This approach is suitable to obtain valid information on the disease status of a population at a given time. But an important disadvantage is the resource consumption especially for rare diseases where large sample sizes are necessary because of the low expected prevalence.

Passive surveillance stands in contrast to active surveillance because animals are only tested if they show clinical symptoms and if those symptoms are detected and reported to the authorities [3]. Passive surveillance is ongoing and not restricted to a certain time frame. It is cost-saving because resources are only needed if disease is suspected. A non-negligible disadvantage is the potential for under-reporting and therefore failure to provide reliable information on the actual disease status of a population. Despite this, passive surveillance plays an important role in early warning systems, especially for rare events like emerging infectious diseases, but it is not yet routinely integrated into the assessment of disease surveillance systems.

According to Martin et al. [4], the sensitivity of a SSC is described as the probability that the SSC will give a positive outcome, given that the disease is present at or above a certain level. A lot of factors influence the sensitivities of passive SSCs, such as the probability of infected animals showing detectable clinical signs, the disease awareness (DA) of persons responsible for reporting, and the sensitivities of the applied diagnostic tests. Therefore, it is difficult to estimate and objectively quantify the probability of detecting cases through passive surveillance, and to be able to evaluate the contribution of this SSC to the whole surveillance system's performance.

One way of objectively analyzing and quantifying the process from clinical manifestation to disease detection is through the use of stochastic scenario tree modelling [4,5]. With this approach, a surveillance process is depicted step by step, and the probability of a particular scenario of case detection can be quantified.

In this article, we describe how we quantified and evaluated the quality of abortion testing (ABT) as a passive SSC for the situation of freedom from *Brucella melitensis *(*Bm*) in Switzerland (CH) and for a hypothetical disease free situation in Bosnia and Herzegovina (BH). *Bm *is a relevant pathogen requiring surveillance because it causes huge economic losses in livestock production, and it is of major concern in human health [6]. Furthermore, brucellosis is classified as a 'neglected zoonosis' by the World Health Organization [7].

## Methods

### Scenario tree approach and surveillance system component

The use of scenario trees for the evaluation of SSCs has been described by several authors in recent years [4,5,8]. A scenario tree is a chronology of events that describes a certain surveillance process step by step. In case of passive surveillance, it describes the process from disease manifestation with detectable clinical signs through to disease notification, reporting and testing of suspect cases. Martin et al. [4] described a stochastic scenario tree modelling approach combining various data sources for the documentation of freedom from disease. This approach integrates all factors influencing infection and detection. Consequently, the population under surveillance can be stratified into sub-groups reflecting their different relative risks for infection and probabilities of detection. Parameters specific to different sub-strata are taken into account when depicting the scenario tree of an SSC. Therefore, the sensitivity of the SSC can be assessed for individual risk regions or population strata. However, within a population, risk factors for infection do not influence the sensitivity of a passive SSC because the whole population is passively surveyed through animal caretakers [4].

The sensitivity of a SSC depends on the level of disease in the population. According to Martin et al. [4], this value is called design prevalence, and it describes the prevalence assumption for which the sensitivity estimate of the SSC is valid. The design prevalence level is a crucial part of the whole scenario tree analysis; i.e. the same SSC may have a high sensitivity for detecting disease present at high prevalence, but a lower sensitivity for the detection of low prevalence disease.

The scenario tree methodology, in its current form, can only be applied in disease free situations where all processed units give negative results. Therefore, the design prevalence is usually set according to international standard design prevalence levels for surveillance [4]. For BH, where *Bm *is endemic, ABT was explored taking into account the population structure and DA levels of the current endemic situation, but modeling a hypothetical disease free situation with an among-flock design prevalence of 0.2% [2] and assuming that all processed units would give negative results. Obviously, the absolute sensitivity value of ABT did not reflect the true situation of BH analyzing the scenario tree model under such assumptions. But because we were interested in analyzing the process of ABT and in identifying the influencing parameters in order to obtain a better understanding of what improves the sensitivity of this SSC, the scenario tree methodology turned out to be a suitable tool for this purpose.

A pivotal parameter influencing the sensitivity of passive SSCs is the DA of persons responsible for reporting. In this context, the term DA implies not only the knowledge about disease characteristics and the vigilance of persons with reporting duties like farmers and veterinarians; it also includes the willingness of those persons to effectively report suspect cases. The willingness to report may depend on disease management factors like compensation payment of culled animals and negative economic consequences due to reporting suspect cases (e.g. ban on animal movement). Consequently, DA is difficult to assess and quantify. Within the scope of this project, the DA of farmers and veterinarians in both countries was assessed qualitatively using expert opinion. The qualitative ranges 'low', 'medium', 'high' and 'very high' corresponded to the quantitative input parameters shown in Table 1[5].

**Table 1:** Disease awareness categories

CATEGORY	DISTRIBUTION	LEVEL
Low disease awareness	*RiskPert *[0.1; 0.2; 0.3]	L
Medium disease awareness	*RiskPert *[0.4; 0.5; 0.6]	M
High disease awareness	*RiskPert *[0.7; 0.8; 0.9]	H
Very high disease awareness	Fixed value [1.0]	VH

Qualitative description of the disease awareness categories used in an assessment of the probability of *Brucella melitensis *case detection using a passive surveillance system component based on abortion testing.

One clinical sign of *Bm *infection is known to be abortion, and abortion testing (ABT) can be regarded as a passive SSC for *Bm*. But the sensitivity and benefit of ABT is difficult to assess and was completely unknown in both countries at the beginning of this study. Therefore, the objective of this study was to quantify and compare the unit sensitivity of ABT and the influential parameters, in the disease free situation of CH and the hypothetical disease free situation of BH. In this context, it must be mentioned that a lot of reasons besides *Bm *can produce abortions in pregnant small ruminants. But within the scope of this project, we assumed that *Bm *was the only reason for an abortion given the flock was infected.

The modeling software used in this work was Microsoft Excel and Palisade @RISK , and we ran the simulations with 5,000 iterations each. The most influential parameters on the scenario tree model outputs were identified evaluating the results of the sensitivity analysis, i.e. the regression coefficients of each input value, given in the @RISK output report.

The reference population in both countries consisted of all sheep and goat flocks having at least one female animal > 12 months. All analyses were based on the flock being the basic surveillance unit. The time frame for our analysis was set to one year and the input parameters used are shown in Table 2.

**Table 2:** Input parameters

**Probabilities of detection**	**Name**	**Value**	**Source**
Proportion of female animals in small ruminant flock in CH and BH	*PrFem*	*RiskPert *(0.90; 0.96: 0.98)	[19]
Proportion of pregnant animals in flock in CH and BH	*PrPreg*	*RiskPert *(0.70; 0.90; 0.95)	[19]
Probability that an infected pregnant female will abort	*Abort*	*RiskPert *(0.187; 0.56; 0.70)	[20-22]
Probability that farmer calls veterinarian in CH (= low DA)	*FCallsVCH*	*RiskPert *(0.10; 0.20; 0.30)	Personal experience DC Hadorn
Probability that veterinarian takes sample in CH (= medium DA)	*SamplCH*	*RiskPert *(0.40; 0.50; 0.60)	Personal experience DC Hadorn
Probability that farmer calls veterinarian in BH (= medium DA)	*FCallsVBH*	*RiskPert *(0.40; 0.50; 0.60)	[19]
Probability that veterinarian takes sample in BH (= medium to high DA)	*SamplBH*	*RiskPert *(0.55; 0.65; 0.75)	[19]
Diagnostic test sensitivity in CH and BH	*TSens*	0.95	
Diagnostic test specificity in CH and BH	*TSpec*	1.00	[4]

Input parameters for the stochastic simulation model to quantify the detection performance of abortion testing (ABT) for the surveillance of *Brucella melitensis *(*Bm*) in small ruminants in Switzerland (CH) and Bosnia and Herzegovina (BH).

### Disease status and surveillance for *Brucella melitensis *in Switzerland

CH is officially free from *Bm*, and the last case of *Bm *was reported in 1985 [9]. Brucellosis is a notifiable disease and Swiss farmers are bound to inform the official veterinary service about each abortion in small ruminants. Veterinarians must take abortion samples if more than one abortion has happened within 4 months on the same farm, and the samples are tested for, among other pathogens, *Bm *[10]. Since 1998, an annual random survey has been conducted in small ruminant populations, additionally to disease notification and abortion testing, to substantiate freedom from disease.

The screening test conducted in CH for *Bm *is the CHEKIT^® ^BRUCELLOSE SERUM ELISA (IDEXX/Dr. Bommeli AG). The confirmatory tests used are the Rose Bengal Test (RBT) and the Complement Fixation Test (CFT) [11].

According to the OIE [2], freedom from *Bm *means that the flock prevalence in a country is = 0.2%. Therefore, the design (flock) prevalence (PstarH) within the stochastic scenario tree model for CH was set to 0.2%.

### Disease status and surveillance for *Brucella melitensis *in Bosnia and Herzegovina

The animal health reporting system in BH has indicated a persistent increase in the number of reported outbreaks of *Bm *infection in ruminants, especially sheep and goats, in recent years. Organized control activities for small ruminant brucellosis date from 1948 [12]. Disease detection in BH is provided by annual serological surveys and serological testing of samples obtained from reported clinical suspect cases.

The testing protocol used for *Bm *in BH is the application of RBT and CFT in series.

Due to the limitation of the applied methodology (described above) and our aim to objectively compare passive surveillance between two countries with different brucellosis status, we set the design prevalence of *Bm *in BH to be 0.2% of flocks (PstarH), even though *Bm *is currently endemic in BH. Thereby, we assumed a hypothetical disease free situation for BH, while estimates of DA levels in BH corresponded to the present endemic situation.

### Within-herd prevalence and small ruminant population structure in Switzerland and Bosnia and Herzegovina

The structure of the population to be surveyed influences the effectiveness of surveillance for infectious diseases because typical levels of the within-flock design prevalence (PstarA) are not directly applicable in small flocks [13]. The minimum, non-zero prevalence in small flocks may be greater than PstarA; e.g. the smallest non-zero prevalence in a flock with 4 animals is 25%. Additionally, small flocks play a special role at low prevalence levels, such as at the time of disease incursion into a population [14]. Therefore, a large proportion of small flocks within a population has an impact on the sensitivity of a SSC.

In our stochastic scenario tree model for CH and BH, PstarA was set to 10% having chosen the median value between the assumption of P. Hopp (5%, personal communication) and the value of 15% given by A. Robinson [15]. Therefore, we assumed a within-flock prevalence of one infected animal per flock in flock size category < 20 according to Greiner and Dekker's definition of small flocks, implying small flocks to be those with < (2/PstarA) animals over 12 months of age [13].

**Figure 1:**
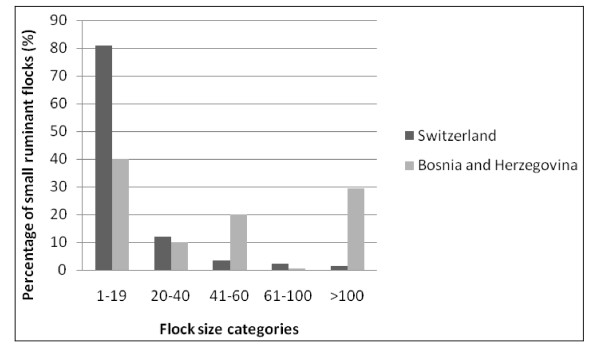
**Small ruminant population structure**. Proportion of small ruminant flocks by flock size categories 1–19, 20–40, 41–60, 61–100 and > 100 animals over 12 months of age in Switzerland (CH) and Bosnia and Herzegovina (BH). The proportions of flock size categories for BH are derived from different sources [17,18].

In order to take into account the influence of the population structure, we stratified the small ruminant population into five flock-size categories, namely flocks with < 20 animals, 20–40 animals, 41–60 animals, 61–100 animals and > 100 animals. In CH, 80.8% of all 23,236 small ruminant flocks consist of 1–19 animals per flock [16]. 11.9% of flocks have 20–40 animals and only 7.3% of all flocks have > 40 animals [16]. The small ruminant population structure in CH is therefore clearly dominated by small flocks (Figure 1).

In BH, no comprehensive animal and farm identification system exists, and therefore no exact data about the population structure is available. For the purpose of this study, the small ruminant population in BH was constructed by combining estimates from different sources on population size and structure [17,18]. The estimated population had a total number of 4,576 flocks of which 40.9% of the small ruminant flocks belonged to the category 1–19, 28.5% of the flocks had 20–60 animals, 0.6% had 61–100 animals and 30% had > 100 animals per flock (Figure 1).

### Scenario tree for abortion testing in Switzerland and Bosnia and Herzegovina

**Figure 2:**
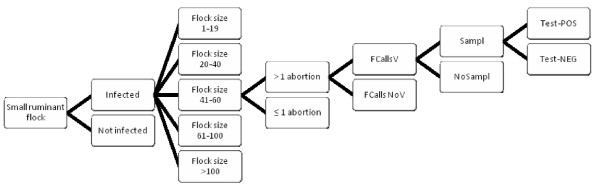
**Basic structure of the scenario tree for abortion testing**. Structure of the scenario tree for the probability of *Brucella melitensis *(*Bm*) case detection based on testing of aborting small ruminants in Switzerland (CH) and Bosnia and Herzegovina (BH). 'FCallsV' means that the farmer calls a veterinarian and 'FCallsNoV' that he does not call a veterinarian. 'Sampl' means that the veterinarian takes samples to test for *Bm *and 'NoSampl' that he does not take samples. 'Test-POS' means a positive test result and 'Test-NEG' a negative test result.

We created a stochastic scenario tree model for the SSC of ABT where the process from disease presence to disease detection was similar in CH and in BH (Figure 2). The input parameters of the scenario tree were also identical for CH and BH with the exception of the small ruminant population structure and the DA in farmers and veterinarians (Table 2).

The selected flock-size categories allowed us to take into account the influence of the proportion of small flocks on the sensitivity of ABT. Additionally, we assumed that one single abortion within a flock would not provoke the farmer to call a veterinarian, i.e. only > 1 abortion happening within the same flock will trigger the detection process (Figure 2).

The number of expected abortions per flock size category, given the flock is infected with *Bm*, was calculated using Equation 1:

(1) *nAbt*_*j*_* = N*_*j *_× *wfPrev *× *PrFem *× *PrPreg *× *Abort *

where:

*nAbt*_*j *_= Number of expected abortions in an infected flock in flock-size category *j*

*N*_*j *_= Number of animals per flock in flock-size category *j*

*wfPrev *= Within-flock design prevalence

*PrFem *= Proportion of female animals per flock

*PrPreg *= Proportion of females that are pregnant within a flock

*Abort *= Probability that an infected pregnant female will abort

The value of the flock size (*N*_*j*_) was simulated according to the population structure using the *Risk Cumulative *function. As suggested by Greiner & Dekker [13], the within-flock prevalence (*wfPrev*) for flocks with < 20 animals was set to 1 infected animal per flock (1/*N*_1–19_). The within-flock prevalence for flocks ≥ 20 animals was equal to PstarA. In order to get integer values for *N*_*j *_and *nABT*_*j*_, the rounding function with zero decimal places was used in Microsoft Excel. Within the scope of this work, we assumed that only flocks with > 1 abortions will be noticed, reported and tested. Therefore, the value for the detection node "Flock with > 1 abortion" was set to 1 when *nAbt*_*j *_was > 1 and 0 when *nAbt*_*j *_was ≤ 1.

The probability that the farmer will report the abortions to the veterinarian is given by the DA of the farmer. Similarly, the DA of the veterinarian influences the probability that *Bm *is suspected and samples are taken. If samples are taken and sent to the laboratory, the quality of the diagnostic test has also an influence on the sensitivity of ABT. Different test protocols are used for the diagnosis of *Bm *in CH and BH as described before. Because we focused on the comparison of ABT in CH with ABT in BH, with special regard to management factors, and not on the influence of laboratory diagnosis, both scenario trees were analyzed for a test sensitivity of 95% and a test specificity of 100% (Table 2). In order to calculate the overall sensitivity of ABT (ABTSe), we first calculated the average probability that an individual flock will test positive (ABTSeU) using the formula described in Martin et al. [4].

The probability that one or more positive flocks out of *n *processed flocks would be detected given PstarH to be ≥ 0.2% (ABTSe), and assuming all units to be independent of each other, was also calculated according to Martin et al. [4]. This formula is depicted in Equation 2:

(2) *ABTSe = 1 - (1 - ABTSeU)*^*n *^

where:

*ABTSe *= Overall sensitivity of ABT

*ABTSeU *= Average ABT unit sensitivity

*n *= Number of processed flocks

ABTSeU is influenced by the population structure and ABTSe by the number of processed flocks *n*. In order to compare the sensitivity of the detection process for ABT between CH and BH independently from the differences in the population structure, and given the same design prevalence of 0.2% of flocks, the average probability was also calculated that an individual *Bm*-infected flock would test positive for each flock size category *j *(PUPos_*j*_) [4].

## Results

The results of the stochastic scenario tree model clearly show that the probability of a *Bm*-infected small flock (1–19 animals) having more than one abortion and therefore being detected as positive through abortion reporting and testing (probability of positive unit PUPos_1–19_) is 0 in both countries (Table 3). Median PUPos_20–40 _is also 0 for both countries. But it is possible to detect *Bm*-infected flocks with ABT even in flock size categories 20–40, as is shown in the values for the 95percentile of the distribution of PUPos_20–40 _(Table 3).

**Table 3:** Results

**Output value**	**Percentiles of output distribution**		
	
	**5%**	**50%**	**95%**
**PUPos in flock-size category 1–19:**			

PUPos_1–19_CH	0.000	0.000	0.000
PUPos_1–19 _BH	0.000	0.000	0.000

**PUPos in flock-size category 20–40:**			

PUPos_20–40 _CH	0.000	0.000	0.112
PUPos_20–40 _BH	0.000	0.000	0.323

**PUPos in flock-size category 41–60:**			

PUPos_41–60 _CH	0.000	0.090	0.126
PUPos_41–60 _BH	0.000	0.305	0.358

**PUPos in flock-size category 61–100:**			

PUPos_61–100 _CH	0.064	0.095	0.127
PUPos_61–100 _BH	0.259	0.307	0.359

**PUPos in flock-size category > 100:**			

PUPos_101–750 _CH	0.064	0.095	0.127
PUPos_101–322 _BH	0.262	0.307	0.359

**ABTSeU**			

ABTSeU CH	6.6E-06	14.6E-06	43.1E-06
ABTSeU BH	2.2E-04	3.2E-04	3.9E-04

**ABTSe**			

ABTSe CH	0.143	0.290	0.635
ABTSe BH	0.631	0.764	0.835

Results for the average probability that an individual *Bm*-infected flock would test positive for each flock size category *j *(PUPos_j_), for the average probability that an individual flock will test positive for *Bm *(ABTSeU) and for the overall sensitivity (ABTSe) of the passive abortion testing system (ABT) in Switzerland (CH) and in Bosnia and Herzegovina (BH). All results base on the true (CH) and a hypothetical (BH) disease free situation.

In CH, the probability of finding a *Bm*-infected flock with ABT in flock size category 41–60 is 9% compared to 30.5% in BH. For flocks with > 61 animals, PUPos in BH is 3.23-times higher with 30.7% compared to 9.5% in CH (Table 3).

According to Equation 2, the sensitivity of ABT is influenced by the number of processed units *n *which corresponds to the total number of small ruminant flocks within each country. The overall sensitivity of ABT (ABTSe) was 2.6-times smaller in CH with a value of 29.0% compared to a sensitivity of 76.4% in BH, although the number of small ruminant flocks in CH is 5.1-times bigger than in BH (Table 3).

In order to correct for the influence of the population size, we compared the average probability that an individual flock will test positive (ABTSeU) in CH and BH to get an objective comparison. Based on this, ABTSeU in CH is 21.9-times smaller than in BH assuming perfect test specificity.

The sensitivity analysis of the stochastic scenario tree model, i.e. the regression coefficients of the @RISK output reports (data not shown), showed that the most influential parameter on PUPos_20–40 _in CH and BH was the flock size. The probability that a *Bm*-infected animal will abort was the most influential parameter on PUPos_41–60 _in both countries. The DA of farmers was the most influential parameter on 'PUPos_61–100 _CH' and 'PUPos_61–100 _BH', as well as on 'PUPos_101–750 _CH' and 'PUPos_101–322 _BH'. The DA of farmers was also an important influential parameter in BH for 'ABTSeU BH' and 'ABTSe BH'. The population structure, i.e. the number of small flocks, was the most influential parameter for 'ABTSeU CH', and the probability that a *Bm*-infected animal will abort was the most influential parameter for 'ABTSe CH'.

## Discussion

The sensitivity analysis of the stochastic scenario tree model for ABT in CH and BH showed that the DA of farmers and the population structure, i.e. the proportion of small flocks, are important parameters influencing the probability of detecting *Bm *with ABT given the disease is prevalent ≥ 0.2%. The results of the model show that ABTSe for the time frame of one year was much higher in BH than it was in CH. In BH, reported outbreaks of *Bm *infections in small ruminants have been increasing in the last few years. Therefore, animal caretakers and veterinarians are likely to be very much aware of and vigilant for this disease. This stands in contrast to the Swiss situation where the last case of *Bm *in small ruminants occurred in 1985. Consequently, the DA in Swiss animal caretakers and veterinarians is assumed to be lower than it is in BH, and the results documented the impact on the detection of cases.

If the disease is not known or neglected in a country, none or only a few suspect cases are reported. But as soon as a disease is introduced, the number of suspect cases increases. This was recently observed in relation to bluetongue (BT) in CH. At the beginning of 2007, the DA for BT was not very high and only 3 suspect cases for BT were reported in the first 5 months of the year. But after the specific information campaigns initiated by the Swiss Federal Veterinary Office to increase the DA in cattle and sheep farmers in June 2007, the number of suspect cases increased to 24 in the next 4 months. After the first BT case detected in CH in October 2007, a further increase in the reporting of suspect cases was recorded (61 suspect cases in November 2007). This shows clearly that DA can be influenced by information campaigns and that management of DA may be an interesting tool for veterinary services.

With regard to *Bm *surveillance in CH, the probability of detecting infected flocks with ABT is currently 0 in flocks ≤ 40 animals and rather low in flocks > 40 animals. This approach is therefore not a very potent instrument to document freedom from *Bm*. But because ABT has comprehensive coverage of the sheep and goat population, it is still a helpful and resource-saving instrument, especially in larger flocks, with regard to an early warning system for *Bm*-incursion. Therefore, it should be considered to improve the sensitivity of ABT by increasing DA of farmers by conducting information campaigns just before the lambing season. To verify this, a simulation was conducted with a medium DA of Swiss farmers and veterinarians, and ABTSe was increased to a medium sensitivity of 0.572 (5percentile = 0.327 and 95percentile = 0.914). Consequently, the overall sensitivity of ABT may potentially be improved by such management actions.

Our model results show that the probability of detecting *Bm*-incursion with ABT depends in general on the DA of farmers, but is negligible in small flocks. Therefore, as a further inference of the model, smaller flocks should be selected for the annual serological survey in order to compensate for a negligible ABTSe in small flock size categories, and information campaigns should especially focus on farmers caring for larger flocks in order to increase DA and hence ABTSe.

The probability of detecting at least one infected flock given the country is infected at the design prevalence (ABTSe) is not relevant for endemic situations like in BH. Nevertheless, the scenario tree model is helpful to evaluate the SSC of ABT for a hypothetical disease free situation in order to find out the most influential parameters and to identify management factors that can be influenced in order to improve the sensitivity of ABT. In BH, the DA of all persons with reporting duties is relatively high due to the presence of *Bm *in human and animal populations. Testing of aborting small ruminants is a common surveillance activity and 'ABTSe BH' is therefore high. This result is not surprising because *Bm *is endemic in BH. But after eradication of *Bm *in BH, it is assumed that the DA will lower over time as fewer and fewer cases occur unless an effort is made to keep DA high by information campaigns.

With regard to the results for PUPos_j _for an assumed within-flock design prevalence (PstarA) of 10%, it can be stated that ABT is only beneficial in flocks > 40 animals in order to support an eradication strategy.

A constraint of the scenario tree approach is the amount and complexity of the input parameters needed. The value of input parameters is often difficult to obtain and, in case of lack of real data, expert opinion may be used to get an approximate value [4]. Nevertheless, this approach offers a methodology for the structured analysis of passive surveillance activities and an objective assessment with regard to its sensitivity. Each step in the whole process of passive surveillance is transparent and the resulting sensitivity of the process being analyzed can be referred to the assumptions made. This transparent way of analyzing and assessing SSCs also offers the possibility of comparing passive surveillance activities between two different countries and in relation to alternative active approaches.

## Conclusion

The probability of *Bm *infection leading to abortion in pregnant animals is relatively high. Therefore, ABT is an effective instrument for the passive surveillance of *Bm*. However, the actual performance of passive surveillance approaches heavily depends on the context in which they are applied. Scenario tree modelling in its current form allows for the assessment and evaluation of the sensitivity of SSCs assuming disease free situation. Potentially, this approach can also be used for endemic situations, but further methodological development is required in this respect.

Despite data gaps, scenario tree modelling is a real opportunity to compare different situations and to explore consequences of changes that could be made.

## Authors' contributions

DCH worked out the scenario tree model for CH, supervised the development of the scenario tree model for BH, analyzed the results and drafted the manuscript. SSH worked out the model for BH. KDCS participated in the design and coordination of the study and contributed to the manuscript. All authors read and approved the final manuscript.
